# A Review on Angiogenesis and Its Assays

**Published:** 2012

**Authors:** Zoya Tahergorabi, Majid Khazaei

**Affiliations:** 1 *Department of Physiology, Isfahan University of Medical Sciences, Isfahan, Iran.*

**Keywords:** Angiogenesis, Assays, Diseases

## Abstract

Angiogenesis or formation of new blood vessels from preexisting vasculature is a key process in some physiological conditions such as wound healing, growth, and action of female reproductive organs. Moreover, disturbance of the mechanisms of physiological angiogenesis has a role in pathogenesis of some diseases in the form of overproliferation of blood vessels such as cancers, psoriasis, arthritis, retinopathies, obesity, asthma, and atherosclerosis or impaired angiogenesis participates in diseases such as heart and brain ischemia, neurodegeneration, hypertension, osteoporosis, respiratory distress, preeclampsia, endometriosis, postpartum cardiomyopathy, and ovarian hyperstimulation syndrome. Research and study in angiogenesis provide a potential to cure a variety of diseases such as cancers or cardiovascular diseases. Thus, in recent years, several methods for evaluation of angiogenesis have been introduced and selecting the most appropriate cure is very important. In this article, first, we briefly reviewed appropriate assays to evaluate therapeutic angiogenesis (clinical manipulation of angiogenesis) and its importance during some clinical diseases and then introduced *in vitro*, *in vivo*, and *ex vivo* assays of angiogenesis besides their benefits and disadvantages. Next, some quantitative techniques for assessing angiogenesis have been discussed.

## Introduction

Angiogenesis plays a crucial role in organogenesis and advanced embryonic and fetal development (1). It is the formation of new blood vessels from preexisting ones (2) that prepares oxygen and nutrients for the cells and removes waste products (3).

In adult organisms, angiogenesis is necessary for wound healing, growth, and action of female reproductive organs including ovulation, follicular development, corpus luteum formation, progesterone release, endometrial growth, regression and repair during the menstrual cycle, and formation of a fully vascularized tissue for implantation and placentation during pregnancy (4-7). 

## Angiogenesis, vasculogenesis, arteriogenesis

Angiogenesis is the formation of new blood vessels from preexisting ones (2). It is mediated via two distinct pathways: splitting and sprouting. Excess in microvascular shear stress leads to the intra luminal splitting of a microvessel linear into two vessels, but tissue hypoxia triggers sprouting angiogenesis and budding of a new capillary sprout laterally from a preexisting vessel (8, 9). Vasculogenesis is *de novo* formation of blood vessels in the embryo that when blood vessels arise from endothelial cell progenitors (angioblasts) (10). It is completed with formation of primary vascular plexus (11). Arteriogenesis is the organization of mature arteries from preexisting arterioles after arterial obstruction. It is able to fully compensate a blocked artery but there is not angiogenesis. Arteriogenesis and angiogenesis are regulated by distinct stimuli. For example, increased shear stress through collateral vessels is an initial stimulus for arteriogenesis while angiogenesis begins with tissue hypoxia/ischemia (12, 13).

## Mechanisms of angiogenesis

In the vessels, there are two main cell types: endothelial and mural cells. Mechanisms of angiogenesis are the study of regulation of biological activities and interactions of these cells with each other (14).

Angiogenesis consists of three stages: the first, selection of some endothelial cells namely "tip cells" inside the capillary to begin angiogenic expansion. ״Tip cells״ have a master role while new vessels grow. These cells react to the angiogenic factor VEGF-A (vascular endothelial growth factor)_. _Therefore, VEGF-A empowers ״tip cells״ for invasion and migration. Selection of "tip cells" is controlled by Notch family receptors (heterodimeric proteins) and their transmembrane ligands DLL4 (Delta like ligand 4) (15). Expression of DLL4 and its Notch receptors are activated by interaction of VEGF with endothelial cells (16). In mammals, there are four Notch receptors and five ligands (jagged 1, jagged 2, DLL1, DLL3, and DLL4) (17). Following function of VEGF, ״tip cells״ orient in the VEGF gradient which is mediated by the interaction of VEGF-VEGFR-2 (vascular endothelial growth factor receptor 2); also ״tip cell״ migration is regulated by the interaction of VEGF-VEGFR-2. The second stage, migration, proliferation of endothelial cells and tube formation, is also mediated by interaction of VEGF-A and VEGFR-2 (18). The third stage, maturation of newly formed vessels, consists of inhibition of endothelial proliferation and migration of new capillaries, stability of already existing new vascular tubes (fusion of the newly formed vessels with others), and role of mural cells (pericytes and vascular smooth muscle cells) (19, 20). Pericytes are in direct communication with endothelial cells to form capillary walls (21). Role of pericytes in formation of walls of newly formed vessels is mainly mediated through PDGF-B (platelet-derived growth factor β) and its receptor, PDGFR-B (platelet-derived growth factor receptor) (22). Similar to VEGFR receptors, PDGFRs are transmembrane proteins that their intracellular region contains tyrosine kinase domain but against VEGFR receptors, the extracellular region of PDGFRs is not seven pass (23).

## Regulation of angiogenesis

Hypoxia upregulates the expression of different genes participant in various steps of angiogenesis such as VEGF, angiopoitin-2, and FGF (fibroblast growth factor) (24). Angiogenesis is mediated by balance and interplay between numerous "pro- and anti-angiogenic" factors (25, 26). Below, some of the most important factors are mentioned.

## Angiogenic factors

*VEGF*

VEGF is one of the most potent angiogenic factors. It is a member of the VEGF family including VEGF-A, VEGF-B_, _VEGF-C_, _VEGF-D, and VEGF-E. VEGF-A gene undergoes alternative splicing and produces 6 isoforms of VEGF-A: VEGF-A121_, _VEGF-A145, VEGF-A165, VEGF-A183, VEGF-A189, and VEGF-A206 that are different in their properties and functions (27). VEGF-A has 3 receptors VEGFR-1 (FLT-1), VEGRR-2 (FLK-1/KDR), and VEGFR-3 (FLK-4).

VEGFR-1 has the highest affinity for VEGF and PLGF (placental growth factor), VEGFR-2 binds to VEGF-A. VEGF-C and VEGF-D both bind to VEGRR-2 and VEGFR-3 (26). There are originally VEGFR-1 and VEGRR-2 in blood vascular system while VEGFR-3 is found in the lymphatic endothelium (28). The effects of VEGF on proliferation and migration are mainly carried out by VEGFR-2 (27). In comparison with normal thyroid tissue, in benign and malignant human thyroid tumors, an increase in the expression amounts of the corresponding messenger RNA of VEGFR-3 gene receptor is observed in the adenomas tissue. On the other hand, during tumor development an alteration in the ratio of VEGFR receptors can be observed (29).

*Angiopoietins*

Angiopoietins are paracrine growth factors. They are ligands for tyrosine kinase receptor Tie-2 but one has not been demonstrated for closely related receptor Tie-1. Interaction of angiopoietin-1 with Tie-2 on endothelial cells is necessary for maturation and stabilization of the developing vasculature (30). However, angiopoietin-2 is an antagonist of angiopoitin-1 that can block angiopoietin-1- induced autophosphorylation of Tie-2 in endothelial cells. In transgenic mouse experiments, overexpression of angiopoietin-2 led to a fatal phenotype similar to that of angiopoietin-1 and Tie-2 knockout mice (31).

*EGF and TGF-α*

EGF (epidermal growth factor) and TGF-α (transforming growth factor-α) are mitogenic for endothelial cells and increase angiogenesis in *in vivo* model. Both bind to the EGF receptor (32). Studies have been demonstrated that the EGF receptor signaling pathway in cancer cells cause cell proliferation increase, angiogenesis, and metastasis besides decreased apoptosis (33). Moreover, stimulation with EGF dose-dependent manner increases VEGF in various prostate cancer cell lines (34).

*FGF*

There are 20 distinct FGF and four different tyrosine kinase receptors (FGFRs) (35). FGF-1 (acidic FGF) and FGF-2 (basic FGF) are part of the first growth factors that stimulate angiogenesis. Both FGF-1 and FGF-2 are bounded to all four FGF receptors and are mitogenic for endothelial cells, fibroblasts, and many other cell types. FGF-2 increases the expression of angiogenic factor VEGF and proteases for example urokinase-type of plasminogen activator. Wound healing is normal in FGF-1 knockout mice whereas FGF-2 and FGF1/FGF2 knockout mice show delay in wound healing likely because FGF-2 regulates sprouting of new vessels at sites of tissue repair (36, 37). 

## Anti-angiogenic factors

*Angiostatin*

Angiostatin is derived from plasminogen spanning the first 4 kringle domains (38). Angiostatin suppresses proliferation, migration, differentiation, and tube formation of *in vitro* endothelial cells and potently inhibits angiogenesis. Systemic administration of angiostatin trough mechanisms consist of binding of the subunits of ATP synthase on the cell surface of endothelial cells might inhibit proliferation and can potently block neovascularization and growth of tumor metastasis (27).

*Endostatin*

Endostatin is derived from enzymatic degradation of collagen XVIII (39). It suppresses migration and proliferation of endothelial cells and increases apoptosis (40). Angiostatic effects of endostatin have been shown by experimental studies that mice with full thickness skin wounds were treated systemically with endostatin that was found to have no effect on vessel density. However, ultrastuctural analysis showed severe abnormalities in the newly vasculature consisting of narrowing or even closing of the wound vessels and irregular luminal surfaces of vessel wall (41).

*Thrombospondin*

The TSP_S_ (thrombospondins) are a family of glycosylated extracellular matrix proteins (42). Two members of the TSP family, TSP-1 and TSP-2, have angiostatic effect. TSP-1 can prevent *in vivo* neovascularization in the cornea pocket assay and inhibits *in vitro* migration and proliferation of endothelial cells and also blocks tube formation. Although there is high structural homology between TSP-1 and TSP-2, the effects of TSP-2 on angiogenesis are different from TSP-1 (43). When TSP-2 with squamous cell carcinoma was injected into the dermis of nude mice, the inhibition of tumor growth was significantly stronger than TSP-1, besides the density and the diameter of tumor vessels were remarkably decreased (27).

## Angiogenesis in diseases

"The angiogenic switch" is a term that implies the balance between angiogenic and angiostatic factors. Disturbance of this balance leads to a growing list of diseases (44) characterized by the over proliferation of blood vessels consisting of hypertension (45), cancers, psoriasis, arthritis, diabetes (46, 47), obesity, asthma, and atherosclerosis. Moreover, defect in angiogenesis can cause heart and brain ischemia, neurodegeneration, hypertension, osteoporosis, respiratory distress, preeclampsia, endometriosis, postpartum cardiomyopathy, and ovarian hyperstimulation syndrome (48). 

*Angiogenesis and cardiovascular disease*

Cardiovascular disease is the most common cause of death in Iran and is the cause for one-third of deaths. Hypoxia in regulating tumor angiogenesis mimics its role in angiogenesis in progression of lesion formation atherosclerosis (49). Intimal thickening of vessels induced by hypercholesterolemia or injury decreases supply of oxygen and nutrients to the media and neointima of vessels through excess distance from either the lumen or adventitial vasovasorum (a microvasculature in adventitial layer of large arteries). A hypoxic region is formed in interior of the artery that stimulates formation of HIF-1α (hypoxia-inducible transcription factor) that in turn induces expression of VEGF and other angiogenic factors. VEGF increases plaque growth through oxygen supply to the media and neointima (24). Plaques contain a lipid core separated from the vessel lumen by thin fibrotic cap and have high macrophage numbers (50). Monocytes/macrophages release mitogenic, proinflammatory, and prothrombotic factors that enhance formation of atherothrombotic lesions (51).

In relation to topic of the gene therapy in angiogenesis or therapeutic angiogenesis, many studies are in progress for the gene and protein delivery of two cytokines, VEGF and FGF, to treat CAD (coronary artery disease) and PAD (peripheral arterial disease) which are atherosclerotic vascular diseases (52). Laham *et al* carried out a double-blind, placebo-controlled phase I randomized trial with local perivascular delivery of bFGF on 24 patients undergoing coronary bypass surgery. Patients were placed in three groups including, Placebo, low dose, and high dose bFGF. Three months after the surgery, in the placebo and low dose groups there was not recovery but in high dose group recovery was seen with SPECT scans showing remarkable dissolving of ischemic areas (53).

*Angiogenesis and cancer*

Angiogenesis is necessary for tumor growth and distribution of tumor cells to distant locations. Hypoxia is a critical process for tumor angiogenesis and is carried out primarily by the transcription of hypoxia-sensitive genes and HIF (54). Several mechanisms were considered for vascularization of tumors including endothelial sprouting and bone marrow-derived endothelial cells and so forth.

Endothelial sprouting is a process that is controlled by balance between ״pro- and anti-angiogenic״ factors. Endothelial sprouting is a basic mechanism for tumor vascularization. During sprouting, pericytes detach and blood vessels dilate and the process is under control of VEGF and angiopoietins (55). During bone marrow-derived endothelial cells process, circulating cells in the peripheral blood may participate in vessel formation (56). The bone marrow is a reservoir of circulating endothelial cells and endothelial progenitor cells. Endothelial progenitor cells can differentiate into endothelial cells and/or compose into blood vessels in the adult person (57). Hurwitz *et al* published considerable trials to show effectiveness of anti-angiogenic drugs in cancer treatment. The study has directed first-line therapy of 815 patients with metastatic colorectal carcinoma. The humanized, recombinant, anti-VEGF antibody bevacizumab (AVASTIN) (5 mg/kg every 2 weeks) or placebo was added to standard first-line chemotherapy (bolus 5-fluorouracil 500 mg/m^2^, leucovorin 20 mg/m^2^, and irinotecan 125 mg/m^2^ every 4-6 weeks). The total response rate was 44.9% in the bevacizumab group in camparison with 34.7% in the placebo group (58).

Some compounds were approved as anti-angiogenic for treatment of solid tumors, such as bevacizumab (AVASTIN) that is a monoclonal antibody that inhibits VEGF-A and has been approved in the USA and EU for metastatic breast, lung, and colon cancer (59). Sunitinib (SUTENT) is a multitargeted inhibitor of VEGF, PDGFR, and FLT-3 that has been approved for advanced RCC (renal cell cancer) (60). Sorafenib (NEXAVAR) is a multitargeted inhibitor of VEGF, PDGFR, and RAF kinase that has been approved in the USA and EU for HCC (hepatocellular carcinoma) and advanced RCC (61). 

*Angiogenesis and preeclampsia*

Impaired angiogenesis is involved during preeclampsia. Preeclampsia is characterized by hypertension and proteinuria after 20 wk of gestation. Whereas untreated can lead to eclampsia, a disease that elicit maternal, fetal, and neonatal morbidity and mortality (62). Several ״pro- and anti-angiogenic״ proteins are produced in the placenta (63) that onset in pregnancy. Proangiogenic proteins are overexpressed for placental angiogenesis and increase in expression of antiangiogenic factors toward the last pregnancy likely in preparing for delivery (64). VEGF as well as its receptors includes VEGFR-1 (FLT1) and VEGFR-2 (KDR) up-regulated efficiently by hypoxia (65) that produces defective trophoblast remodeling of the uterine spiral arteries (66). Anti-angiogenic molecule, soluble Fms-like tyrosine kinase-1 (SFLT-1), is produced by alternative splicing of the FLT-1 gene that can be bound to VEGF and leads to endothelial dysfunction and prevent angiogenesis in placenta. Clark *et al* have demonstrated by *in situ* hybridization that trophoblasts express the SFLT-1mRNA that increases toward the end of pregnancy. Western blotting of villus-conditioned media has shown the production of SFLT-1 (67). Recent data have demonstrated that abundant production of the anti-angiogenic proteins such as SFLT-1 and/or increment in antiangiogenic factors toward the end of pregnancy happens too soon are the likely mechanisms for the syndrome of preeclampsia (64). 

*Diabetic retinopathy*

One of the basic problems of diabetes is diabetic retinopathy. Diabetic retinopathy leads to blindness in diabetic persons (68). Chronic hyperglycemia provokes the endothelial dysfunction, causes the thickening of capillary basement membranes, enhances vasopermeability, and increases ROS (reactive oxygen species) production (69). The increased oxidative stress enhances inflammation that in turn causes hypoxia (70). Hypoxia up-regulates HIF-1α that in turn provokes the production of VEGF. VEGF is a crucial growth factor in genesis of the diabetic retinopathy, with creation of extra vascular permeability and angiogenesis (71). VEGF acts through two tyrosine kinase receptors, VEGFR-1 and VEGFR-2 and VEGFR-2 levels are increase in diabetic retinopathy. VEGFR-2 is the main receptor of the angiogenic and proliferative effects of VEGF (72). Miller and colleagues demonstrated that the level of VEGF-A in the ocular tissue of eye is in relation to new vessel formation (73). Moreover, trials by Funatsu and colleagues showed that modulation balance of angiogenesis inducers and inhibitors in controlling neovascularization is important in diabetic retinopathy (74). On the other and, in hyperglycemic states in diabetic retinopathy, aldose reductase (derived from the non-enzymatic glycation of proteins) reduces glucose into sorbitol due to slow reaction sorbitol into fructose and its aggregation may cause osmotic cellular damage and endothelial dysfunction (72). 

*Angiogenesis and arthritis*

RA (Rheumatoid arthritis) is a chronic inflammatory autoimmune disease (75). There are many similarities in pathophysiology between solid tumors and inflammatory diseases, e.g., RA, consists of interference cytokines and hypoxia in creation of angiogenesis (76). RA may begin at any age from early life to ninth decade with peak time 35-45 years old that is the working ages (77). Angiogenesis is known as a significant process in the progression and preservation of RA disease and the potent angiogenic factor, VEGF, plays a critical role (78). In RA synovial fluid is filled with neutrophils and excess in volume leads to joint swelling and pain (79). The synovial hyperplasia that is the result of inflammation in RA can lead to an extra need for oxygen and nutrients in the inflamed synovium. The hypoxic microenvironment of synovium induces expression of hypoxia sensitive angiogenic factors, e.g., VEGF and interaction with inflammatory and cytokines factors and signaling pathway such as NFκB, TNF-α, and IL-6 (80). A recent study using ultrasonography to assess synovial thickening has demonstrated proliferation and remarkably lower synovial fluid pO_2 _levels in RA compared with non-RA patients (81). In RA synovium, HIF-1α isoforms (HIF-1α, HIF-2α) are expressed. Therefore, inhibition of the hypoxia/HIF-1α pathway in diseases such as cancers and RA can be an alternative therapeutic target (82). 

## In vivo angiogenesis assay

*Matrigel plug assay*

The Matrigel plug assay is considered as a fast screening test for stimulators and inhibitors of angiogenesis (83). Matrigel is an extract from EHS (Engelbreth-Holm-Swarm) mice sarcoma. It consists of extracellular matrix components and growth factors. Matrigel is stored at -20 ^°^C and before use is thawed at 4 ^°^C that becomes liquid and makes a solid gel (Matrigel plug) at 37^°^C (body temperature of mice). Pro-angiogenic and anti-angiogenic substances are added to Matrigel and are injected subcutaneously into the ventral region of animals (84). Angiogenic response in the Matrigel plug depends on the characteristics of the animals under experiment and location of injection. Young mice (6 months old) create less new blood vessels in comparison with older ones (12 to 24 months) (85). Moreover, lower angiogenic response is observed if Matrigel is injected into the dorsal surface of the animal instead of ventral side in the groin area close to the dorsal midline. After 7-10 days, Matrigel plug is removed and new blood vessels formation in Matrigel can be observed (84, 86). Since the Matrigel plug at first is avascular, any vessel that is formed in Matrigel plug is easily is distinguishable (85). Vessel formation can be quantified by measuring the hemoglobin (Drabkin method) and is confirmed by histological staining (84, 86). 

**Figure 1:**
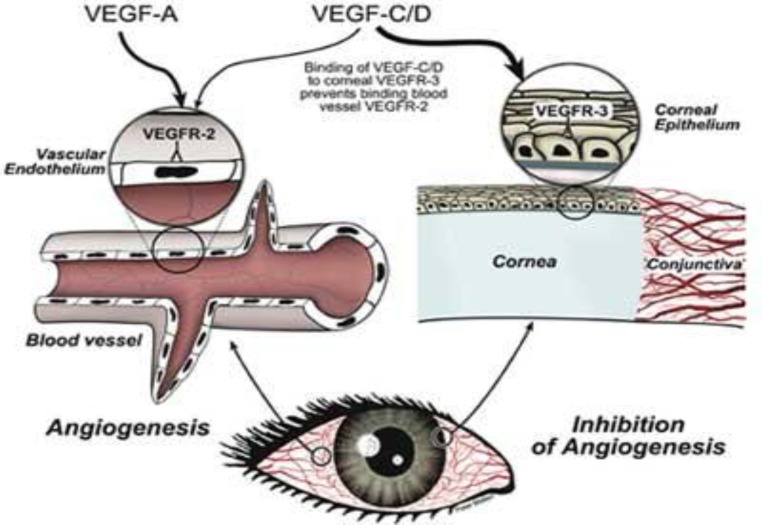
Corneal angiogenesis assay    (90)

*Corneal angiogenesis assay*

Cornea of rat is 250-255 μm thick whereas in mice it is slightly less thick. The corneal stroma that forms about 80% of the corneal thickness in rats contains wide sheets of parallel collagen fibers dipped in an extracellular matrix (87). 

This method has been used originally in rabbits but adapted for rats and mice (88). In this method, a pocket is created in the cornea and the test substances are placed into micropockets that stimulate or inhibit the formation of new blood vessels. Since cornea of eye is an avascular tissue, vessels that form in the cornea can be easily seen and this is one of the considerable advantages of the corneal angiogenesis assay, (Figure 1) but the space available for placing test substances is limited and may create inflammatory reactions (89). In this method, microvessel density can be detected through immunohistochemistry using endothelial markers and vascular response can be quantified by computer image analysis following perfusion of the cornea with India ink (85).

*Chick chorioallantoic membrane (CAM) assay*

**Figure 2:**
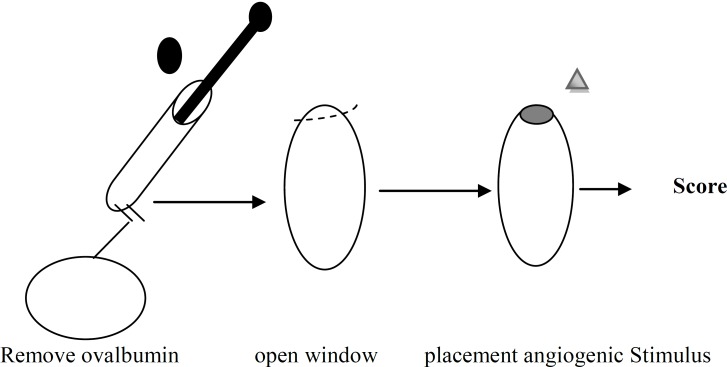
CAM assay    (93)

In this method, test substances (both stimulators and/or inhibitors) are placed on the extra embryonic membrane through a window that was created in the eggshell chick embryos on days 7-9. Then window was sealed, eggs were reincubated and angiogenesis was studied after suitable incubation time (91) (Figure 2). Although CAM assay is a relatively easy and inexpensive *in vivo* assay but there are some problems in this assay including CAM undergoes rapid morphological change and thus masks distinguished new capillaries from existing ones. While, the 7-9 days old CAM often has an inflammatory response to various substances which can prevent the recognition of new vasculature (88). Quantifying the CAM angiogenesis response can be done by scoring the extent of vascularization on a graded scale of 0-4. Moreover, in another method, gelatin sponges with an angiogenic stimulator or inhibitor are placed on the growing CAM. Growth of blood vessels can be accomplished vertically into the sponge and the edge between the sponge and the surrounding mesenchyme and number of blood vessels are counted 4 days after placement (85, 92).

*Hindlimb ischemia assay*

**Figure 3:**
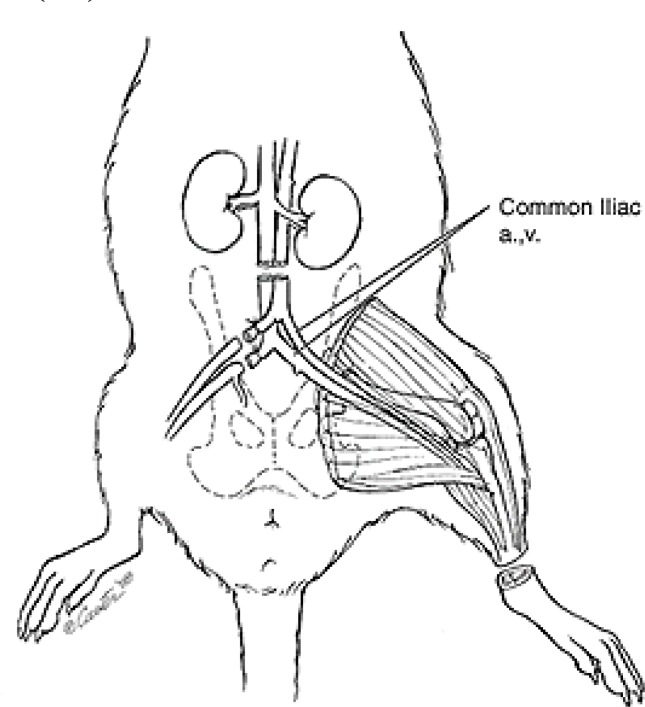
Hindlimb ischemia in rat    (97)

Unilateral hindlimb ischemia is the animal model of peripheral arterial disease that has been used extensively to study vascular remodeling (13, 89). Hindlimb ischemia in a mouse with vessel exposure begins by incision on the skin overlying the middle portion of the hindlimb, where continuous proximal end of the femoral artery and the distal portion of the saphenous artery are tied and the arteries beside side-branches are cut (Figure 3). The mechanism of arteriogenesis (hemodynamic change) is notable at the tied location whereas angiogenesis (hypoxia/ischemia) predominates in the ischemic distal location (94). Neovascularization is determined by increasing endothelial cell proliferation and capillary density (95). Capillary density is the most common method for histological measure of angiogenesis. Since muscle atrophy in hindlimb ischemia is a common event, counting of capillaries per muscle fiber may be more proper than the number of capillaries per square millimeter (96).

## In vitro angiogenesis assays

There are difficulties in relation to endothelial cells in *in vitro* assay including, they are not homogeneous, and there is a high degree of heterogeneity among endothelial cells. For example, from the viewpoint of vessel phenotype, there is a continuous vessel that endothelial cells line the complete internal surface of the vessel wall, whereas in a fenestrated vessel, endothelial cells are permeable and small openings present among endothelial cells. In sinusoidal vessels, many fenestrations are among endothelial cells and have large lumen. On the other hand, there is disparity in endothelial size, shape, and complexity of junctions and this heterogeneity in endothelial cells are as a result of their placement in different locations within the body which must be considered when endothelial cells are selected for *in vitro* assay (98).

*Endothelial cell proliferation assays*

There are two major kinds of proliferation assays: determination of net cell number and study of cell-cycle kinetics.

Net cell number can be carried out by a hemocytometer (using a light microscope) or an electronic counter such as coulter counter. The hemocytometer is time-consuming, more prone to sampling error, and can only count high density of cells whereas coulter counter can count low density of cells and is a more rapid and safer method. Another method commonly used for proliferation assay is MTT [3-(4, 5-dimethylthiazol-2-yl)-2, 5-diphenyltetrazolium bromide] that is degraded by a mitochondrial dehydrogenase enzyme to produce purple/blue formazan crystals which aggregates in living cells. These crystals are dissolved by the addition of DMSO and the solubilized formazan product is evaluated using an Elisa plate reader (88). 

DNA synthesis for studying cell-cycle kinetics is to use BrdU (Bromodeoxyuridine) which competes with thymidine for participation into the DNA in the S-phase of the cell cycle. BrdU can be determined by immunocytochemistry in individual cell level or by Elisa for the cell population. Direct cell-cycle examination using DNA-binding molecules and flow cytometric analysis can be an alternative method for analyzing proliferation. In this method, at first, BrdU participate into cellular DNA and then saturation staining of propidium iodide is carried out. A correlation between the BrdU content of the cells with propidium iodide shows the cell-cycle distribution, proliferation state, and a measure of apoptosis that is one of the advantages of this method (99).

*Endothelial cell migration assays*

Endothelial cells during process of angiogenesis break down the basement membrane with release of proteolytic enzymes such as matrix metalloproteinases (MMP_S_) and migrate in response to a gradient of angiogenic factors including VEGF. Modified Boyden chamber assay is the most frequent method to assess endothelial cell migration. In this method, endothelial cells are placed on top of a filter containing 8 µm diameter pores which usually are coated with fibronectin, collagen protein, or Matrigel and a test angiogenic factor (attractant substrate) is placed in the lower chamber, then endothelial cell migration is carried out in direction of attractant substrate (100). High sensitivity to small differences in concentration gradients, high reproducibility, and short duration are benefits of this method. Technical difficulties in setting up the assay, preservation of the trans filter gradients for prolonged periods of time, and inability to observe cell migration during experiment are considered disadvantages of this assay (101). Another method for cell migration assay is measurement of an angiogenic response as angiogenic factors stimulate cell movement that was seen in the Boyden chamber assay but more accurately was seen in phagokinetic track assay. In this method, colloidal gold-plated coverslips are used as substrate for the movement of cells. This assay has been modified to large scale screening using beads attached to the bottom of 96-well plates. The main disadvantages of this assay is migration of cells on an alien substrate was completed that was not seen *in vivo* assay also, in each assay a few number of endothelial cells are examined (88).

*Endothelial cell differentiation assays*

These assays are the measurement of the ability of endothelial cells to form three-dimensional structures (tube formation). The formation of tight junctions between the endothelial cells in the developing tubules is studied by electron microscopy (98). Rate of differentiation of endothelial cells depends on the type of matrix used. For example, cell proliferation with only occasional tubule formation is created with plating of endothelial cells on collagens I and III but culturing on collagens IV and V lead to extensive tubule formation with only minimal proliferation. Matrigel-derived from the mouse Engelbreth-Holm-Swarm sarcoma is the most powerful matrix for tubule formation that begins to form within 1 hr and is completed within 12 hr following endothelial cell plating. Thus, mechanisms implicated in the differentiation of endothelial cells to form tubules are at least partly dependent to the matrix (102). Moreover, use of Matrigel, collagen, and fibrin clots is feasible to generate 3D tubule formation assay to prepare *in vitro* model of tubulogenesis that more precisely mimics* in vivo* model (103). Another form of tubule formation assay is to use culture of endothelial cells with stromal cells with or without an extracellular matrix (ECM). The stromal cells that can be used include fibroblasts, smooth muscle cells, and blood vessel explants. When human fibroblasts are co-cultured with endothelial cells, the fibroblasts with secretion of matrix components empower the endothelial cells to form tubules that have lumen. However, this method is less well-characterized (104). Another alternative method of tubule formation is using alkaline phosphatase-coupled anti-CD31 antibody linked to a soluble chromogenic substrate and then using Elisa (105). 

## Comparison of in vitro and in vivo assays

1. *In vitro* assays recognize direct effects on endothelial cell function while *in vivo* assays involve multiple cell types.

2. *In vitro* assays analyze isolated processes of angiogenesis consisting of proliferation, migration and differentiation, or tubule formation of endothelial cells. Whereas *in vivo* assays angiogenesis as a whole.

3. *In vitro* assays technical skill in animal handling is not needed compared with that of *in vivo* assays.

4. *In vitro* assays are less expensive than *in vivo* assays.

5. *In vitro* angiogenesis assays often can be quantified more easily (of course interpretation of *in vitro* assays must be carried out with extreme caution because endothelial cells are used in isolation) in comparison with *in vivo* assay (106, 107). Some of advantages and disadvantages of angiogenesis assays are illustrated in Table 1.

## Ex vivo assays (organ culture)

Identification of the fact that angiogenesis *in vivo *involved endothelial cells in addition to their surrounding cells led to assess angiogenesis by organ culture methods (88). These assays mimic *in vivo* situations because they contain surrounding nonendothelial cells (such as smooth muscle cells and pericytes) and a supporting matrix.

*The rat aortic ring assay*

 In this method, at first, thoracic aortas are removed. Then, peri-aortic fibroadipose tissue is carefully separated in culture dish containing ice-cold serum-free medium without damage to the aorta wall. Finally, the aorta is cut into 1 mm long rings and aortic rings are placed in the 24 well plate. The rings are immersed in Matrigel and then the microvessel outgrowth is monitored over a period of 10-14 days (108). There are problems in aortic ring assay, e.g., explants from different animals give variable results because of species-specificity. Moreover, all organ culture assays are described for non-human tissues that makes doubtful applicability of these assays (109). 

*Chick aortic arch assay*

A modification of rat aortic ring assay is the chick aortic arch model (110) with the advantage of rapid result as it takes 1-3 days. At first, the aortic arches are isolated from 12-14 chick embryos (that are derived from growing embryos and endothelial cells have many properties of microvascular endothelial cells) and are cut into 1 mm rings and are cultured in well plate containing Matrigel. Endothelial cell outgrowth in both the aortic ring and the chick aortic arch cultures can be quantified by the use of fluorescein-labeled lectins, e.g., BSL-I and BSL-B4 or by staining the cultures with labeled antibody to CD31 (111).

## Quantitative angiogenesis assay

Angiogenesis can be quantified by invasive and noninvasive methods. Measurement of morphometric parameters by light or electron microscopy of vasculature and histological examination of tissue sections stained with endothelial antibody or perfusion with intravascular markers such as colloidal carbon, India ink, and high molecular weight tracers are categorized as invasive methods (112). Noninvasive methods include: MRI (magnetic resonance imaging), functional CT (computed tomography), and PET (positron emission tomography) scanning. Noninvasive methods in comparison with invasive methods have a much lower resolution and generally cannot expose the vessels of the microcirculation. Many noninvasive techniques basically are developed for human, that at present have been scaled to imaging of mouse (113).

**Table 1:** Advantages and disadvantages of angiogenesis assays

Type of assay		Subtype of assay	Specific assay	Benefits	Disadvantages
*In vitro*	Endothelial cell proliferation assay		MTT	-Measurement of proliferation of living cells and indication of cell number	-Drugs may affect cellular metabolism that can lead to increase or decrease in cell number
*In vitro*	Endothelial cell proliferation assay		BrdU	-Measures of the total DNA per cell - Give cell-cycle information -Measures cell apoptosis	-Does not measure toxicity of drugs
*In vitro*	Endothelial cell migration assay		Boyden chamber	-High sensitivity to small differences in concentration gradients	-Technically is difficult
*In vitro*	Endothelial cell differentiation assay		Matrix assays	-Using computer assist for processing images of complete wells	-Lumen may not be formed -Other non-endothelial cell types may participate in tube formation on Matrigel
*In vitro*	Endothelial cell differentiation assay		Co-culture of endothelial cells with stromal cells	-Co-culture stromal cells such as fibroblasts with endothelial cells enable the endothelial cells to form tubules containing lumen by secretion matrix components from fibroblasts	Time-consuming
*In vivo*	-		CAM assay	-Relatively inexpensive assay -Appropriate for large-scale screening	-Placing under morphological rapid change that makes identification of new capillaries from preexisting ones difficult
*In vivo*	-		Corneal angiogenesis assay	-Identification of new blood vessels is easy	-Relatively expensive assay -Is not suitable for large-scale screening
*In vivo*	-		Matrigel plug	-Technically is not difficult -Quantification can be accomplished by measuring the amount of hemoglobin in the plug	-Analysis is time-consuming -Expensive assay
*In vivo*	-		Unilateral hindlimb ischemia assay	-Suitable method mimics peripheral artery disease -Nonexpensive method	-Variations in hindlimb injury and blood flow recovery
*Ex vivo*	Organ culture assay		The rat aortic ring assay	-Mimics *in vivo* situation	-Used tissues derived from growing embryos that undergo proliferation before explantation are not good representative of the nonproliferative endothelial cells existing in *in vivo* assay
*Ex vivo*			Chick aortic arch assay		

 *Capillary density estimation*

Measurement of microvascular density in histological sections is still the gold standard for quantification study of the angiogenesis. The density of blood vessels in histological sections is greatly affected by section thickness (114). In this method, tissues are fixed in buffered formalin, progressively are dehydrated in increasing percentages of ethyl alcohol, embedded in paraffin, sectioned at 5 µm thickness, are deparaffinized and stained with anti-CD31 antibody. Then, they carry out continuous incubation with biotinylated anti-rat IgG, avidin-biotin peroxidase, and diaminobenzidine substrate. Finally, sections are counterstained with H&E (hematoxylin and eosin) and are observed under light microscope. The capillaries are counted and expressed as number of capillaries per mm^2 ^(115).

*Assessment of hindlimb ischemia*

Capillary density is the most common histological measurement of angiogenesis in hindlimb ischemia. Since muscle atrophy is common in hindlimb ischemia model, counting the number of capillaries per muscle fiber is likely more accurate than the number of capillaries per mm^2 ^(96). pO_2 _measurement is carried out using a pO_2_ probe that is placed in the ischemic muscle by EPR (electron paramagnetic resonance) (116, 117). The hindlimb function can also be expressed by forced swimming test to define the functional capacity of the ischemic hindlimb. In this method, animal is placed in a water-filled tank to swim. Functional muscle activity is determined as the ratio of number of active stokes/min of the ischemic limb (during 3 successive periods) to the healthy limb (118). Lower extremity blood flow visualization can be carried out with laser doppler perfusion imaging. Micro CT and MRI can also be used to visualize collateral vessels (119).

*Hemoglobin and FITC-dextran determination in Matrigel plug*

Determination of hemoglobin concentration in Matrigel plug is an indicator of the number of blood vessels in the plug. In this method, Matrigel plug is dissected from the mouse and isolated of any surrounding tissues. Plug is weighed and homogenized in 1 ml deionized H_2_O for 5-10 min on ice. Then it is placed at 10000 rpm on microcentrifuge for 6 min at 4ºC and the derived supernatant is collected for hemoglobin measurement based on manufacturer’s protocol. Supernatant is mixed with Drabkin’s reagent and retained at room temperature for 15-30 min. One-hundred µl of this mixture is placed in a 96-well plate. Absorbance is measured with Elisa plate reader at 540 nm. Hemoglobin concentration is compared with a standard curve. Calculated values are normalized by dividing the hemoglobin percentage by the plug weight and are expressed as g/dl hemoglobin per milligram Matrigel (120).

FITC-dextran (fluroscein isothiocyanate dextran) with molecular weight of 200 kd is used as the index of functionality of neovessel in Matrigel plug. In this method, 20-30 min before sacrifice of mouse and removing of Matrigel plug, 0.2 ml of 25 mg/ml FITC-dextran in PBS (phosphate-buffer-saline) is injected into the lateral tail vein of each mouse and allowed to circulate for 20-30 min. Then the mouse is killed and Matrigel plug is removed and separated from surrounding connective tissues and placed into tubes containing 1 ml of 1:10 dispase and is incubated in the dark in 37ºC shaker overnight. The following day, plugs are homogenized and centrifuged at 3000 g for 10 min and its supernatant remained in dark for analysis of fluorescence reading. Moreover, blood samples are collected by cardiac puncture into heparinized tubes, centrifuged after their collection and plasma is separated and retained from light at 4ºC. Results are expressed as ratio of Matrigel plug fluorescence per plasma fluorescence (121).

## Conclusions

On the basis of this review, it is apparent that no single angiogenic assays (*in vivo* or *in vitro*) can elucidate the entire process of angiogenesis whereas can be useful combination this assays. Many technological advances have occurred recently in the field of angiogenesis and quantification of newly formed microvessels in laboratory animals that mimic various human diseases processes.
